# Impact of vitamin D and vitamin D receptor activator in diabetic nephropathy

**DOI:** 10.3389/fcdhc.2025.1537336

**Published:** 2025-03-11

**Authors:** Merita Emini Sadiku

**Affiliations:** ^1^ Medical Faculty, University of Prishtina “Hasan Prishtina”, Prishtina, Kosovo; ^2^ Clinic of Endocrinology, University Clinical Center of Kosova, Prishtina, Kosovo; ^3^ Policlinics “endoCLINIC Medical & Laboratory”, Prishtina, Kosovo

**Keywords:** diabetic nephropathy, vitamin D, vitamin D receptors, vitamin D receptor activator, renoprotection

## Abstract

Vitamin D is a hormone which is involved in many physiological processes in addition to bone metabolism and the muscular system. Based on several animal and human studies, it has been established that vitamin D plays an important role in the development of diabetic nephropathy (DN). DN is a frequent and severe chronic microvascular complication of diabetes mellitus (DM). As such, DN and cardiovascular complications are considered the main risk factors for the death of patients with DM. Recent studies have shown the renoprotective effect of VD and its receptor activators (VDRAs or VD analogs based on its effect on endothelial function, preservation of podocytes, anti-inflammatory effect, and direct influence on the renin-angiotensin aldosterone system. The renoprotective effect of VD has been shown to potentially delay the onset of DN, which is the main cause of end stage renal diseases (ESRD). The impact of vitamin D on the recovery of already existing kidney damage is debatable and doubtful. Increasing evidence has shown that the VD/VDR interaction possesses a series of renoprotective effects in DN patients based on the anti-proteinuric, anti-fibrotic, and anti-inflammatory effect, as well as the preventive effect of podocyte damage. Based on this important renoprotective effect, important data for therapeutic and effective methods for DN have also been presented. It was performed a structured search of published research literature for several databases regarding the impact of VD on the pathophysiology of DN as well as its therapeutic implications in terms of renoprotection of VD and VDRA in animal research and human clinical research as RCT, observational studies, systematic reviews and meta-analyses over the last decade.

## 1 Introduction

Diabetic nephropathy (DN) is one of the most common and severe microvascular complications of diabetes mellitus (DM), significantly contributing to increased mortality in patients with DM. Chronic Kidney Disease occurring among people with diabetes is considered common, morbid, and costly (1). The International Diabetes Federation estimates that 537 million people were living with diabetes in 2021, with an expected increase to 784 million by the year 2045 (2). As the prevalence of diabetes has increased, the prevalence of DN has grown proportionally (3). The prevalence of CKD among people with diabetes is >25%, and it has been estimated that 40% of people with diabetes develop CKD during their lifetime (3).

To date, several key mechanisms are known to explain the development and progression of DN, including oxidative stress, angiotensin II (Ang-II), and inflammatory processes (4). Investigating the role of inflammatory mechanisms in the development and progression of DN not only enhances our understanding but also identifies novel therapeutic targets for the development of anti-inflammatory therapeutic strategies (4).

Patients with VD deficiency are at an increased risk for developing DN. Recent basic and clinical studies have demonstrated that VD and its analogs exhibit kidney protective effects, such as anti-proteinuria, antifibrosis, anti-inflammatory, and prevention of podocyte damage by binding to the vitamin D receptor (VDR). However, the specific mechanisms by which VD and VDR influence various pathological processes in DN remain unclear. This review aimed to summarize the current research on the role of the VD/VDR signaling pathway in DN and explore the clinical therapeutic potential of VD and VDRAs in renoprotective processes.

## 2 VD and VDRAs

VD is a fat-soluble prohormone crucial for calcium and bone metabolism. In addition, VD affects various tissues of the body, including the kidneys. The two primary sources of VD are diet (20%) and skin exposure to sunlight (80%), the latter initiating the synthesis of several VD-related cascades in the body. VD undergoes two hydroxylation processes to become its active form: the first process occurs in the liver, converting VD to 25(OH) D3, and the second process takes place in the kidneys, resulting in 1, 25-dihydroxyvitamin D [1,25(OH) 2D3]—also known as calcitriol. The renal production of 1,25 (OH)2D3 in the body is normally regulated by the levels of parathyroid hormone (PTH) in the plasma and the levels of calcium and phosphorus in the serum. VD is transported in the bloodstream by binding to VD-binding protein (VDBP). VD deficiency has reached epidemic proportions globally and is associated with several health problems, including defects in bone mineralization (such as osteoporosis and osteomalacia), an increased risk of DM (5), immune defects (6), and cardiovascular disease (7).

Several studies have found that VD levels are lower in patients with DN. According to the Endocrine Society, VD deficiency is defined as a 25(OH)D3 level of less than 20 ng/mL (50 nmol/L) (8). The uptake and activation of VD depends on the formation of the VDBP/25(OH) D3 complex, its filtration, and subsequent reabsorption in the renal proximal tubular cells. In DN, studies have shown increased urinary elimination of VDBP, leading to elevated concentrations of VDBP in the urine (9). The biological effects of VD are mediated through the VDR, which is present in a variety of tissues and cells, such as the intestines, bones, parathyroid glands, reproductive system (10), and kidneys (11, 12).

The interaction between VD and VDR in these diverse tissues explains the broad range of symptoms and signs that can arise from VD deficiency. Numerous studies suggest that the VD/VDR axis is linked to the development of various diseases.

In the kidneys, VD with VDR specifically target proximal and distal tubular epithelial cells, glomerular parietal epithelium, collecting duct cells, mesangial cells, podocytes, and the juxtaglomerular apparatus (10, 13). As is well established, the kidneys play a crucial role in VD metabolism by regulating the reabsorption of calcium and phosphate and controlling the synthesis of its active form. The multiple effects of the VD/VDR signaling pathway include the maintenance of mineral homeostasis, the regulation of inflammation and immune responses, and potential therapeutic effects in cancer (14, 15).

## 3 Role of VD in the pathogenesis of diabetic nephropathy

In the kidneys, VD plays a crucial role in maintaining podocyte health, preventing epithelial-to- mesenchymal transition (EMT), preventing inflammation, and suppressing the expression of the renin gene by regulating the renin-angiotensin-aldosterone system (RAAS) (16). DN, also referred to as a diabetic kidney disease (DKD), is characterized by increased urinary albumin excretion, decreased glomerular filtration rate (GFR), or both (2). Approximately 40% of patients with DM who develop DN, are at risk of progressing to end-stage renal disease (ESRD) and an increased risk of developing cardiovascular diseases (17, 18).

DN is a serious problem that unfavorably affects human health. Mounting evidence from animal and clinical studies has suggested that VDRAs has beneficial effects on various renal diseases including DN mainly by reducing albuminuria.

Hyperglycemia is the primary driver of intracellular metabolism dysregulation, inflammation, enhanced cell apoptosis, and tissue fibrosis (19). High glucose induces oxidative and nitrosative stress in many cell types causing the generation of species such as superoxide, nitric oxide and peroxynitrite and their derivatives. Herby, the role of these species in high glucose-mediated apoptotic cell death is relevant to the complications of diabetes such as neuropathy, nephropathy and cardiovascular disease. Hyperglycemia causes activation of several proteins involved in apoptotic cell death, including members of the caspase and Bcl-2 families (20).

**Figure 1:**
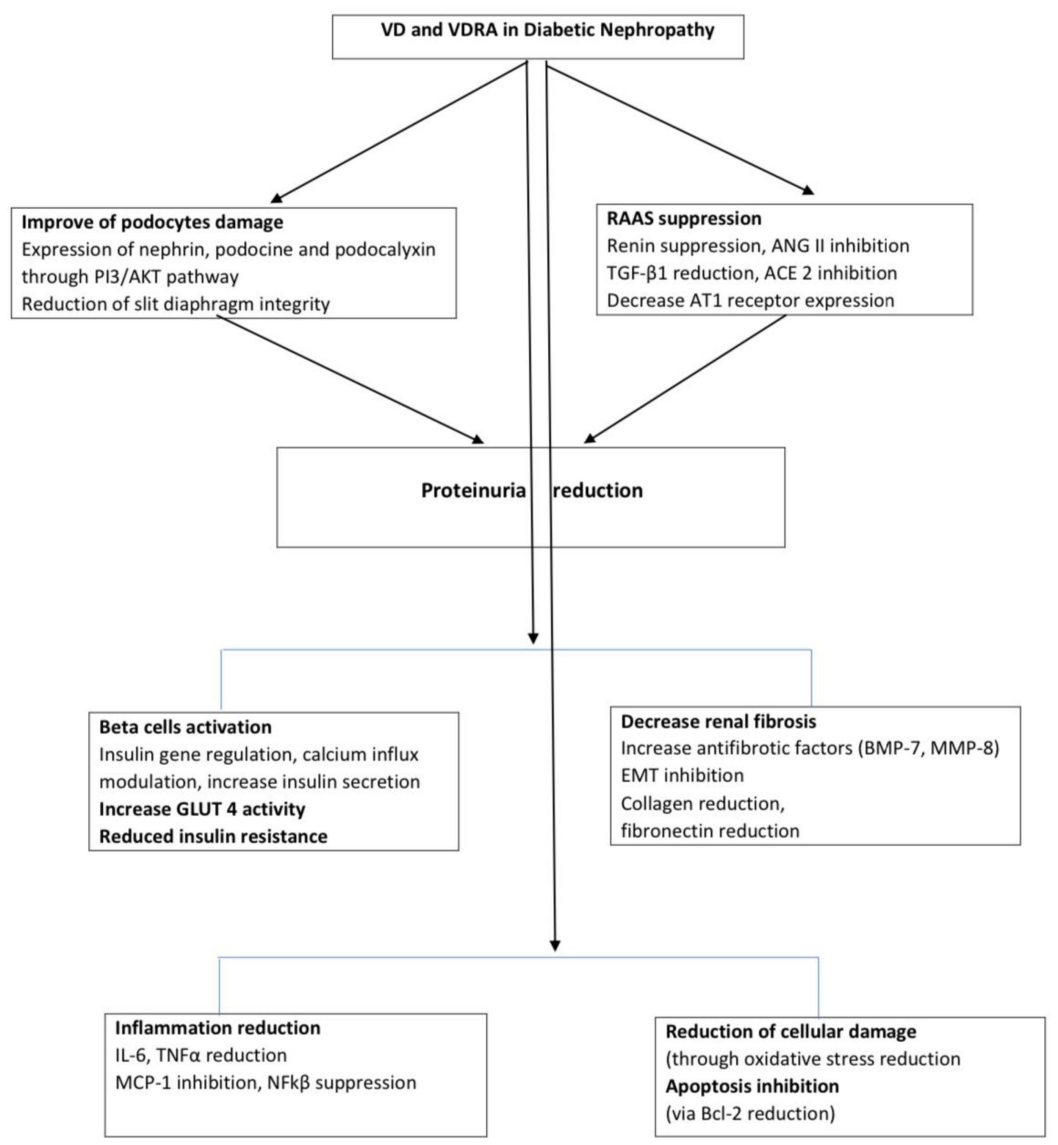
Pathways of VD effects on DN. DN, diabetic nephropathy; VD, Vitamin D; VDRA, vitamin D receptor activator; ANG II, angitensin II; TGF-β, Transforming growth factor-beta; PI3/AKT, phosphatidylinositol 3 kinase; ACE2, angiotensin converting enzyme 2; AT1, Angiotensin II receptor type 1; EMT, Epithelial-to-mesenchymal transition; BMP-7, bone morphogenetic protein 7; MM8, matrix metalloproteinase-8; IL-6, Interleukin 6; TNFα, tumor necrosis factor α; MCP-1- Monocyte chemoattractant protein-1, NFkβ- Nuclear factor kappa-light-chain-enhancer of activated B cells, Bcl2, B-cell lymphoma 2.

The role of VD in the pathogenesis of DN is multifaceted, particularly in terms of podocyte injury, RAAS alteration, inflammatory responses, and insulin resistance, as discussed below and illustrated in the **Figure 1**.

### 3.1 Association of VD with podocyte injury and renal tubular lesions

DN is often considered a podocyte disease (21). Podocyte injury is a primary cause of proteinuria in DN. Podocytes form the outermost layer of the glomerular filtration barrier. Nephrin, plays a renoprotective role through the intracellular signaling pathway (22); podocin; and podocalyxin are essential components of the slit diaphragm. The active form of VD, 1,25(OH)2D3 or calcitriol, could promote the expression of nephrin, which is crucial for maintaining the structure of the slit diaphragm formed between adjacent crossed foot processes. Proteinuria in DN results from the presence of proteins and other large molecules in the urine due to damage to the slit diaphragm. This damage exacerbates the dysfunction of the kidney’s filtration structure, leading to the worsening of proteinuria. In this way in DN, the expression of nephrin, podocin, and podocalyxin is reduced in podocytes, while their urinary secretion is increased, which correlates with the presence of proteinuria (23).

1,25(OH)2D3 exerts a direct protective effect on the kidneys by significantly reducing the levels of interleukin (IL)-6 and IL-8 in proximal tubule epithelial cells and modulating the effector T-cell activity (24). These findings highlight the critical role of VD in renal tubules, with the VD/VDR complex offering significant protection against the progression of DN.

Recent studies have explored the role of the VD/VDR signaling pathway for its renoprotective effects in the kidneys against the development and progression of DN. In animal studies have shown that paricalcitol, a VDRA, significantly improved urinary albumin excretion in 10-week-old db/db mice after just four weeks of treatment (21). Furthermore, both calcitriol and paricalcitol have been shown to reactivate and preserve the expression of specific podocyte components, restoring the selective permeability of the renal filtration barrier and preventing proteinuria (13, 22). Calcitriol has also been shown to increase the level of Bcl2, exerting an anti-apoptotic effect induced by hyperglycemia, while reducing the pro-apoptotic effect of Bad and Bak in cultured mouse podocytes (24). The effect of VDRAs on the reduction of proteinuria in DN is also attributed to the inhibition of acetylheparinase gene promoter activity in podocytes (25). Additionally, VDRA has been shown to enhance nephrin expression, improving podocyte damage and consequently preventing proteinuria in DN through the PI3K/AKT pathway (22).

### 3.2 VD and suppression of the renin-angiotensin system

The RAAS has been widely investigated in patients with DM due to its role in intraglomerular hemodynamics and structural changes in both the glomerulus and tubulointerstitium. The inhibition of RAAS, which plays an important role in the progression of renal disease, can inhibit the progression of chronic kidney diseases, which are characterized by decreased proteinuria and preserved renal function (26). RAS activation increases the angiotensin II (ANGII) levels, which stimulate renal transforming growth factor-beta 1 (TGF-1) production in the mesangium and epithelial tubular cells along with other cytokines and growth factors in renal cells (endothelin-1, monocyte chemoattractant protein-1 (MCP-1), and IL-6), normal T cells expressed and presumably secreted, and osteopontin) (16).

VD suppresses renin biosynthesis and acts as a potent negative regulator of the RAAS, as observed in various models of kidney diseases (27–30). A study by Riera et al. demonstrated that paricalcitol, a synthetic analog of VD, inhibits angiotensin-converting enzyme 2 activity in non-obese diabetic mice and provides protection against DKDs (27). In cell cultures, 1,25(OH)2D suppressed renin gene transcription through a mechanism involving the VDR (28). In another study, VD was shown to suppress RAAS and TGF-β activation in a hyperglycemic environment, thereby preventing tubulointerstitial fibrosis (29).

Furthermore, 1.25(OH)2D3 can block the increased activity of intercellular renin, extracellularly released Ang II levels under hyperglycemia conditions, and decrease AT1 receptor expression (30).

### 3.3 VD and insulin resistance

The result of basic and clinical studies revealed that vitamin D deficiency is a crucial factor that may accelerate insulin resistance formation. The 1,25(OH)_2_D exerts an effect on insulin sensitivity via regulation of extracellular Ca^2+^ concentration and its flux through cell membranes (31). It has been also observed that vitamin D deficiency contributes to increasing Ca^2+^ concentration that may decrease GLUT-4 activity leading to insulin resistance (32). Additionally, the VDR response element was identified in the insulin gene promoter in pancreatic cells, suggesting that calcitriol directly stimulates insulin release (33). It was shown that 1,25(OH)_2_D might activate Peroxisome proliferator-activated receptor delta (PPAR-δ) that in turn improved insulin sensitivity (34). PPAR-δ decreases FFAs-induced insulin resistance in skeletal muscle (35).

### 3.4 VD in interstitial fibrosis

Studies have found that the expression of VDR in renal tissue is significantly decreased in mice with renal interstitial fibrosis, highlighting the importance of VDR in kidney function. The downregulation of VDR can be reversed with the treatment of VDAs, such as paricalcitol. Moreover, the therapeutic effect of paricalcitol on renal interstitial fibrosis is closely associated with its ability to regulate VDR expression. It was demonstrated that 1,25(OH)2D3 can inhibit the progression of DN fibrosis by inhibiting the production of fibronectin and the activation of TGF-β and RAAS in high glucose-treated mesangial cells (29). Additionally, 1,25(OH)2D3 can directly block TGF-β-induced EMT and extracellular matrix proteins in cultured tubular cell by antagonizing the nuclear factor kappa light-chain enhancer of activated B-cell (NF- κB) activity. 1,25(OH)2D3 can improve renal fibrosis by reducing the expression of collagen and other key profibrotic factors, while increasing the expression of antifibrotic factors, such as bone morphogenetic protein 7 and matrix metalloproteinase-8 (13). VDR also directly inhibits the expression of Snail and stimulates E-cadherin expression in primary tubular cell cultures, thereby preventing EMT. These findings confirm the protective role of VDR in inhibiting renal fibrosis (36, 37).

### 3.5 VD and inflammation

Inflammation is considered a major pathological factor in the onset and progression of DN. It contributes to the pathogenesis of DN through various proinflammatory cytokines, including IL-1β, IL-6, IL-18, MCP-1, tumor necrosis factor-α, and inflammatory mediators such as fibronectin and renin in cultured podocytes (13).

Emerging evidence suggests that VDR activation plays an anti- inflammatory role by inhibiting the activation of NF-κB in tubular and mesangial cells (25, 38). In cultured mouse podocytes the activation of VDR by VD inhibited TGF-β1 expression in podocytes; on the contrary, VDR suppression promoted the increase of TGF-β1 expression (24).

Paricalcitol and calcitriol, through VDR activation, exert local renal anti-inflammatory effects in DN rats. These compounds have been shown to prevent the increase in MCP-1, IL-6, renin, and fibronectin mRNA expression and the secretion of MCP-1 to the culture media induced by hyperglycemia in cultured podocytes (38).

## 4 Treatment role of VD and VDR activator in diabetic nephropathy

Many efforts have been made to standardize the treatment of patients with DN, with a primary focus on halting the progression of DN. New therapies targeting oxidative stress and inflammation have sparked significant research interest. In managing DN, first of all, maintaining glycemic control is crucial, and the American Diabetic Association recommends an HbA1c level of 7% (39).

With regard to the antidiabetic therapies recommended for DN and maintaining glucoregulation without damaging the kidneys, incretin-related drugs (such as dipeptidyl peptidase 4 inhibiators-DPP4i and glucagon like peptide 1 receptor agonist-GLP-1 RA) are commonly used for glycemic control. Other promising therapies include mineralocorticoid receptor antagonists, endothelin receptor antagonists, and therapies targeting inflammation and free radicals.

Additionally, resveratrol and Nrf2 are gaining attention for DN management. Notably, there is significant interest in VDRAs (paricalcitol and calcitriol). These agents help control proteinuria and have a significant role in blocking the RAAS (40). The renoprotective effect of VDRAs involves the inhibition of podocyte apoptosis and protects against podocyte damage (41). Furthermore, calcitriol has been shown to reduce the levels of fibroblast growth factor 23 (FGF23), which is a risk factor for the development of DN and can contribute to podocyte edema (42). Paricalcitol, a third-generation VD analog widely used to prevent and treat DN, is often used to replace active VD concentrations and provides a negative feedback stimulus to the parathyroid gland, reducing the secretion of PTH. Paricalcitol is preferred over calcitriol as it results in fewer episodes of hypophosphatemia and hypercalcemia (43). In a large-scale RCT (VITAL study), treatment with 2 μg of paricalcitol for 24 weeks in patients with both type 2 DM and DN led to a reduction in residual albuminuria excretion (44). Numerous studies have also confirmed the renoprotective effects of paricalcitol and its ability to reduce albuminuria in patients with DM (45, 46).

Many other RCT and well-designed observational studies have reported the recovery of renal function after VD therapy in patients with DN where except albuminuria reduction, it was also found increased eGFR growth and decreased serum creatinine (48, 49, 51, 53, 54) while some other RCT except albuminuria reduction have reported no significant differences in serum creatinine and eGFR (53). A few studies have shown controversies with no statistically significant benefit of vitamin D therapy on renal function (50, 52). Please refer to **Table 1** for main findings from clinical studies of VD and VDRA on DN (studies with synergical drug combination were not considered in analyses) and **Table 2** for different forms of VD medications used in the treatment of DN.

**Table 1:** Main findings from clinical studies of vitamin D and vitamin D receptor activators treatment in diabetic nephropathy patients.

Study	Design	Population	Intervention	End point Measures	Main Results
Vitamin D and its analogs					
Krairittichai et al. (48) (2012)	Randomized controlled trial	T2DM, UPCR> 1 g/g, eGFR> 15 mL/min/1.73 m^2^ (n = 91)	Oral calcitriol 0.5 μg twice weekly for 16 weeks (n = 46)	UPCR, eGFR	Percent changes in UPCR (−18.7% vs. +9.9%, *p* < 0.01), a 30% or more decrement in proteinuria (43.5% vs. 11.1%, *p* < 0.01), mean eGFR (35.5 vs. 36.9 mL/min/1.73 m^2^, *p* = 0.83) in treatment group vs. placebo.
Liyanage et al. (49)(2018)	Randomized controlled trial	DN, UACR > 30 mg/g, GFR > 30 mL/min (n = 82)	IM vitamin D_3_ 50,000 IU monthly for 6 months (n = 41)	Plasma rennin, UACR, GFR, serum creatinine	Plasma renin (8.83 vs. 14.19 pg/mL, *p* = 0.006), urine albumin (117.6 vs.163.4 mg/g, *p* = 0.001), serum creatinine (0.77 vs. 0.87 mg/dL, *p* = 0.10), GFR (93.7 vs. 83.9 mL/min, *p* = 0.03) in treatment group vs. placebo.
Bonakdaran et al. (50)(2012)	Cross-sectional study	T2DM, albuminuria (n = 119)	Oral calcitriol 0.5 μg daily for 8 weeks (n = 43)	Albumin excretion rate, FBS, HbA1C, hs-CRP, lipid profile	Albumin excretion rate (73.1 to 57.1, *p* = 0.22) in vitamin D insufficiency/deficiency group.
Mao et al. (51) (2014)	Prospective observational study	T1DM, microalbuminuria (n = 31)	Oral calcitriol 0.25 μg daily for 6 months (n = 24)	Urinary albumin excretion, urine MCP-1, urine TGF-β, TNF-α, IL-6, C-peptide, HbA1C, serum calcium, phosphorus, PTH, 25(OH)D levels, CRP	Urinary albumin excretion (127.05 to 104.81 μg/mg, *p* < 0.05), urine MCP-1/creatinine (99.38 to 89.57 ng/mmol, *p* < 0.05), urine TGF-β/creatinine (79 to 72.33 ng/mmol, *p* < 0.05), TNF-α (57.7 to 47.09 pg/mL, *p* < 0.05), IL-6 (44.04 to 39.88 pg/mL, *p* < 0.05) in vitamin D insufficiency/deficiency group.
Thethi et al. (52) (2015)	Randomized controlled trial	T2DM, eGFR 15–59 mL/min/1.73 m^2^ (n = 60)	Oral paricalcitol 1 μg daily for 3 months (n = 27)	IL-6, hs-CRP, TNF-α, MCP-1, ICAM-1	No significant differences in outcomes between the paricalcitol and placebo groups.
Barzegari et al. (53) (2019)	Randomized controlled trial	DN, albuminuria > 30 mg/day, GFR < 60 mL/min (n = 50)	Oral calcitriol 50,000 IU weekly for 8 weeks (n = 25)	Blood/urine parameters, oxidative/anti-oxidative markers, lipid profile	Urine protein (233.96 vs. 319.91 mg/dL, *p* = 0.006), serum creatinie (1.13 vs.1.31 μg/mL, *p* = 0.59), GFR (46.96 vs. 46.46 mL/min/1.73 m^2^, *p* = 0.81) in treatment vs. placebo.
Liyanage et al. (54) (2021)	Randomized controlled trial	DN, UACR > 30 mg/g, GFR > 30 mL/min (n = 85)	IM vitamin D3 50,000 IU monthly for 6 months (n = 42)	BMD, BMC	Total body BMD, total body BMC and BMDs of spine, femoral neck and total hip regions (+2.0%, +2.2%, +1.8%, +2.1% and +2.6%, *p* < 0.05 for all within-group differences) in treatment group.

(Adapted with permission from the article: Huang HY, Lin TW, Hong ZX, Lim LM. Vitamin D and Diabetic Kidney Disease. Int J Mol Sci.2023 Feb 13;24 (4):3751. doi: 10.3390/ijms24043751. PMID: 36835159; PMCID: PMC9960850).

T2DM, type 2 diabetes mellitus; T1DM, type 1 diabetes mellitus; DN, diabetic nephropathy; UPCR, urine protein to creatinine ratio; eGFR, estimated glomerular filtration rate; UACR, urine albumin to creatinine ratio; IM, intramuscular; HS-CRP, high-sensitive C-reactive protein; FBS, fasting blood sugar; HbA1C, glycated hemoglobin; MCP-1, monocyte chemoattractant protein-1; ICAM-1, intercellular adhesion molecule-1; TGF-β, transforming growth factor-β; TNF-α, tumor necrosis factor-α; IL-6, interleukin 6; BMD, bone mineral density; BMC, bone mineral content; PTH, pararthyroid hormone.

**Table 2:** Therapeutic implications of vitamin D and vitamin D receptor activators in diabetic nephropathy.

Generic Name	Dosage Forms and Common Formulations
Inactive precursors	
Cholecalciferol	Oral capsule (2000 IU; 5000 IU; 10,000 IU; 50,000 IU)
(Vitamin D_3_)	Oral tablet (400 IU; 1000 IU; 5000 IU; 10,000 IU; 50,000 IU)
	Oral liquid (10,000 IU/mL; 25,000 IU/mL; 50,000 IU/mL)
	Sublingual liquid (5000 IU/mL)
	Injectable solution (300,000 IU/mL; 600,000 IU/mL)
Alfacalcidiol	Oral capsule (0.5 mcg; 1 mcg))
(1 α-hydroxy vitamin D_3_	Oral drops (2 mcg/mL)
	Injectable solution (2 mcg/mL)
Active forms or analogs	
Calcitriol	Oral capsule (0.25 mcg; 0.5 mcg)
(1,25-dihydroxyvitamin D_3_)	Oral liquid (1 mcg/mL)
	Injectable solution (1 mcg/mL)
Paricalcitol	Injectable solution (2 mcg/mL; 5 mcg/mL)
	Oral capsule (1 mcg; 2 mcg; 4 mcg)

IU, international unit.

A systematic review and meta-analysis assessed the effects of VD supplements on renal function, inflammation, and glycemic control in patients with DN. The analysis showed that VD supplements, including calcitriol, alfacalcidol, and 1,25(OH)2D3, are beneficial in reducing the levels of 24-hour urine protein and inflammation markers, such as high-sensitivity C-reactive protein (hs-CRP). However, these supplements have no significant effects on hemoglobin A1c, serum creatinine, estimated GFR, and glycemic control indices in patients with DN (47).

Krairittichai et al., in their open-label RCT, revealed that percent changes in urine protein creatinine ratio (UPCR) from baseline to the last evaluation in the calcitriol (0.5 ug twice weekly) for 16 weeks and control groups were -18.7% and + 9.9% (p < 0.01) respectively (48). In another double-blind RCT, patients with DN were administered 50,000 UI per month of intramuscular calcitriol for 6 months, resulting in a significant reduction in urinary albumin creatinine ratio (49).

## 5 Conclusions

Animal and clinical studies have demonstrated that VD and VDRA not only regulate calcium and phosphorus levels they also improve the clinical outcomes of DN. Their benefits include reducing proteinuria, exerting anti-fibrotic and anti-inflammatory effects, and preventing podocyte damages. These properties make VD and VDRA promising options for the prevention and treatment of DN. However, long-term followed-up, RCT of larger sample size, placebo controlled comprehensive outcome measure, are necessary to comprehensively evaluate the effect of VDRA on DN, in order to clarify the real impact of VD on DN including their impact on proteinuria, renal function, progression to ESRD, morbidity, and mortality.
